# Plant-derived extracellular vesicles and nanoparticles: origins, functions, and applications

**DOI:** 10.3389/fbioe.2026.1758558

**Published:** 2026-02-13

**Authors:** Faris Alsaid, Brisa Davila, Baoye He

**Affiliations:** Department of Plant Pathology and Microbiology, Texas A&M University, College Station, TX, United States

**Keywords:** cross-kingdom communications, plant-derived extracellular vesicles (PDEV), plant-derived nanoparticles(PDNP), plant-derived nanotechnology, vesicle biogenesis

## Abstract

Plant-derived extracellular vesicles (PDEVs) and plant-derived nanoparticles (PDNPs) are emerging plant-based nanomaterials with growing relevance in biotechnology, agriculture, and health. Although often grouped together, they arise from distinct origins: PDEVs are actively secreted vesicles with selective cargo loading, whereas PDNPs form during tissue disruption and reflect the lipid-metabolite composition of plant biomass. This review summarizes recent progress in distinguishing these systems, including advances in biogenesis, isolation, biomarkers, and functional characterization. We highlight mechanistic insights into PDEV-mediated cross-kingdom RNA communication in plant immunity and the strong translational potential of PDNPs in oral drug delivery, immunomodulation, and microbiome regulation. Remaining challenges include standardization, scalable purification, and deeper mechanistic clarity. By clarifying their differences and complementary strengths, this review outlines a foundation for developing reliable plant-derived nanovesicle technologies.

## 1 Introduction

Plant-derived nanomaterials have gained attention for their biocompatibility, sustainability, and versatility in biomedical and agricultural applications. Among these, plant-derived extracellular vesicles (PDEVs) and plant-derived nanoparticles (PDNPs) have emerged as two major categories of plant-derived nanomaterials with rapidly expanding relevance in biotechnology, agriculture, and health. Although often discussed together, PDEVs and PDNPs originate from fundamentally different processes. PDEVs are actively secreted vesicles by living plant cells through vesicle trafficking pathways into the apoplastic space or culture medium. In contrast, PDNPs are nanoparticle populations generated during mechanical disruption of plant tissues and encompass a heterogeneous mixture of vesicular and non-vesicular particles, including nanoscale lipid vesicles, fragmented membranes, lipid-protein complexes, metabolite-rich nanoparticles, and other nanoscale assemblies. As a result of PDNP extraction methods, PDNP preparations may include a small fraction of co-isolated PDEVs, whereas the majority of particles are not derived from regulated secretion pathways. Despite these differences, both systems share many key features: biocompatible, enriched in bioactive lipids and metabolites, and capable of carrying functional molecular cargo, making them attractive for translational applications with continued advances in mechanistic understanding.

The growing interest in PDEVs stems from their emerging roles as carriers of small RNAs, mRNAs, proteins and lipids that can modulate biological processes in the interacting organisms (He et al., 2021; Cai et al., 2023; Liu et al., 2024; Wang S. et al., 2024). Recent studies have shown that specific small RNAs can be selectively enriched in PDEVs and delivered into fungal, oomycete and bacterial pathogens, where they suppress virulence-related genes and contribute to plant immunity (Cai et al., 2018; Hou et al., 2019; Ravet et al., 2025). PDEVs have also been shown to respond to environmental stresses and hormonal treatments, with studies demonstrating that abiotic stress, salicylic acid, and other hormone treatments modulate vesicle secretion and cargo composition (Rutter et al., 2022; Koch et al., 2025). Selective loading of small RNA cargoes into PDEVs has been demonstrated, for example, through plant RNA-binding proteins-mediated sorting into PDEVs (He et al., 2021), yet the biogenesis pathways that govern vesicle formation and release in plants are still not fully understood.

PDNPs, on the other hand, have gained attention primarily through their demonstrated bioactivity and utility as delivery vehicles (Huang D. et al., 2025). Nanoparticles derived from edible plants, such as ginger, grapefruit, and strawberry, have shown anti-inflammatory, antioxidant, and regenerative effects in mammalian systems (Yang et al., 2018; Lee et al., 2020; Perut et al., 2021; Zhang et al., 2021). Their stability, oral bioavailability, and ability to encapsulate therapeutic molecules have enabled potential applications in drug delivery, wound healing, modulation of gut microbiota, and immune regulation. Because PDNPs can be produced in large quantities from plant biomass, they offer practical advantages for scalable applications compared to PDEVs. However, their heterogeneity and the lack of defined biogenesis pathways present challenges for mechanistic interpretation and reproducibility.

Despite rapid progress, research on both PDEVs and PDNPs remains at an early stage. Isolation and purification methods vary widely, leading to heterogeneity in particle populations and difficulties comparing results across studies. Although several protein families have recently been proposed as potential PDEV markers, broadly validated and universally accepted markers are still lacking (Rodríguez De Lope et al., 2025), and the composition of PDNPs remains highly dependent on plant species, growth conditions, and extraction protocols. Mechanistic insights into PDEV secretion, uptake, and cargo delivery are limited, and the biological effects of PDNPs often remain correlative rather than causative. These challenges highlight the need for more rigorous methodologies, clearer differentiation between particle types, and systematic studies to establish their biological relevance.

This review aims to more clearly distinguish PDEVs from PDNPs, a distinction that has not always been consistently applied, and to compare their biogenesis, isolation approaches, and molecular composition while highlighting their complementary applications. By integrating current findings and key unanswered questions, we provide a foundation for future research and the development of reliable, application-ready plant-derived nanovesicle technologies.

## 2 Origin of PDEVs and PDNPs

### 2.1 Origin of PDEVs

**FIGURE 1:**
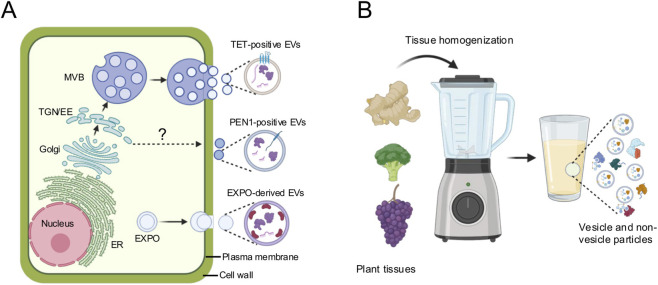
Key Differences Between Plant-Derived Extracellular Vesicles (PDEVs) and Plant-Derived Nanoparticles (PDNPs). **(A)** PDEVs are actively secreted nanovesicles produced by living plant cells through regulated intracellular trafficking pathways. TET-positive EVs are released into the extracellular space by MVB fusion with the plasma membrane and the subsequent release of intraluminal vesicles. The biogenesis pathway of PEN1-positive EVs remains unclear. The EXPO produces EVs by fusion with the plasma membrane to release the inner vesicles into the extracellular space. PDEVs are typically isolated from apoplastic washing fluid, where they appear as relatively uniform, membrane-bound vesicles that selectively carry small RNAs, mRNAs, proteins, and lipids. **(B)** PDNPs are operationally defined nanoparticle populations generated through mechanical disruption of plant tissues, such as homogenization or blending. PDNP preparations consist of a heterogeneous mixture of vesicular and non-vesicular particles, including membrane fragments, lipid-protein complexes, metabolite-rich nanoparticles, and other nanoscale assemblies. As a result of extraction-based methods, PDNPs may also contain a small fraction of co-isolated PDEVs, although the majority of particles do not arise from regulated vesicle biogenesis pathways. Abbreviations: ER, endoplasmic reticulum; EXPO, exocyst-positive organelle; MVB, multivesicular body; TET, tetraspanin; TGN/EE, trans-Golgi network/early endosomes. This figure was created in BioRender.

PDEVs and PDNPs have often been grouped together in the literature and in many cases used interchangeably, yet they originate from fundamentally different processes, making it essential to clearly distinguish between them. PDEVs are naturally secreted extracellular vesicles secreted by plant cells, containing bioactive cargoes like sRNAs, mRNAs, proteins, and lipids for signaling and defense (Figure 1A). It has been experimentally demonstrated that different species of plants, from monocots to dicots, can actively secrete PDEVs. For example, *Arabidopsis thaliana* secretes PDEVs enriched in small RNAs delivered to fungal pathogen *Botrytis cinerea* to suppress infection (Cai et al., 2018; He et al., 2021). Rice and sorghum produce apoplastic EVs containing defense-related proteins that are involved in defense against fungal infection (Chaya et al., 2024; Huang Y. et al., 2025). Alfalfa also secretes EVs detectable in the apoplastic space under the infection of hemibiotrophic fungi *Colletotrichum higginsianum* (Ghosh et al., 2024). Beyond leaf apoplastic fluids, PDEVs have also been detected from root tissues. Tomato hairy root cultures, for instance, secrete PDEVs with demonstrated antifungal activity, and PDEVs isolated from *Salvia dominica* hairy roots exhibit pro-apoptotic activity in pancreatic and breast cancer cells (De Palma et al., 2020; Boccia et al., 2022).

### 2.2 Origin of PDNPs

PDNPs do not arise from an active secretion pathway but are generated during mechanical disruption of plant tissues (Figure 1B). These nanoparticles are commonly prepared by juicing, blending, or homogenizing fruits and vegetables, followed by differential centrifugation. As a result, PDNPs represent heterogeneous populations of lipid-protein complexes, metabolite-rich nanodroplets, membrane fragments, and occasionally PDEVs mixed together. PDNPs have been isolated from a broad range of edible plants: for instance, ginger-derived nanoparticles were found to be enriched in phosphatidic acid (∼47% of total lipids) and used for anti-inflammatory/therapeutic applications (Zhang et al., 2016). Grapefruit-derived lipid PDNPs have been developed for oral drug and siRNA delivery (Wang et al., 2013). Broccoli-derived nanoparticles carry tissue-specific metabolites linked to tryptophan pathways and modulate gut microbial composition and host physiology (Duan et al., 2023). Other vegetable sources such as cabbage, cucumber, as well as fruits like tomato, apple and grape, produce tissue-specific lipid and metabolite profiles in their PDNP fractions (Huang D. et al., 2025). Although PDNPs lack regulated biogenesis, their high yield, biocompatibility, and tissue-specific metabolite profiles make them powerful natural nanocarriers with broad therapeutic and nutraceutical potential.

## 3 Isolation and characterization of PDEVs and PDNPs

### 3.1 Isolation of PDEVs

Because PDEVs and PDNPs originate from fundamentally different sources, the greatest distinctions in their isolation arise at the stage of collecting the starting material. PDEVs must be obtained from extracellular spaces where vesicles are naturally secreted, most commonly from apoplastic washing fluid (AWF) of intact leaves or from cell culture supernatants, which preserve the extracellular origin of the vesicles. AWF-based isolation has been successfully established for *Arabidopsis thaliana*, rice, sorghum, alfalfa, and barley, and ensures minimal cellular rupture when performed with careful vacuum infiltration and low-speed clarification (Cai et al., 2018; Huang et al., 2021; Chaya et al., 2024; Ghosh et al., 2024; Thieron et al., 2024; Huang Y. et al., 2025). Root-derived PDEVs can also be collected from sterile hairy root cultures, such as tomato and *Salvia dominica*, which secrete EVs with antifungal or pro-apoptotic activity (De Palma et al., 2020; Boccia et al., 2022).

### 3.2 Isolation of PDNPs

In contrast, PDNPs are typically isolated from blended or juiced plant tissues. As a result, PDNP preparations represent heterogeneous mixtures of vesicular and non-vesicular nanoparticles, reflecting the composition of the starting tissue rather than defined secretion pathways. Once the starting material is collected, however, the downstream purification workflows for PDEVs and PDNPs are remarkably similar. Both rely on combinations of differential ultracentrifugation, density gradient ultracentrifugation using sucrose or iodixanol, ultrafiltration, or size-exclusion chromatography to enrich nanoscale particles and remove large debris. Differential ultracentrifugation remains the most widely used method but is limited by long processing times and the potential for vesicle deformation at high g-forces. Density gradient separation improves purity by resolving vesicle-like particles from protein aggregates and other co-isolated materials and has been applied to both PDEVs and PDNPs across diverse plant species. More recently, anion-exchange chromatography combined with ultrafiltration or fast protein liquid chromatography (FPLC) systems has been introduced for Brassica and other species, offering enhanced purity, scalability, and preservation of vesicle integrity relative to conventional methods (Zanotti et al., 2025). In addition to these bulk purification approaches, highly specific isolation of PDEV subpopulations can be achieved through immunoaffinity pull-down using antibodies against known EV membrane proteins, such as the Arabidopsis tetraspanin TET8 (He et al., 2021). Although immunoisolation yields substantially lower quantities of vesicles, it provides the highest level of purity and enables the study of defined EV subtypes with minimal contamination, an important advantage for mechanistic and functional analyses.

### 3.3 Characterization of PDEVs and PDNPs

For both PDEVs and PDNPs, characterization methods are largely shared: transmission electron microscopy (TEM) to confirm morphology, nanoparticle tracking analysis (NTA) for size and concentration, dynamic light scattering (DLS) for size and zeta potential, and RNA seq/proteomic/lipidomic/metabolomic analyses to define molecular composition (Rutter and Innes, 2017; Cai et al., 2018; Zhu et al., 2024; Huang D. et al., 2025). Despite recent improvements, challenges remain in standardization, yield variability, and contamination, particularly for PDNPs isolated from high-complexity juices and homogenates. Continued optimization of combined methods will be essential for producing high-purity, application-ready plant-derived nanovesicles.

## 4 Development of novel biomarkers for PDEVs/PDNPs

### 4.1 Biomarkers of PDEVs

Given that many studies use the terms PDEV and PDNP interchangeably, clear biomarker criteria are needed to discriminate *bona fide* extracellular vesicles from other plant-derived nanoparticles. For PDEVs, most work has been done in Arabidopsis and other Brassicaceae species, where the tetraspanins TET8 and TET9, the syntaxin PEN1/SYP121, and EXO70E2 (for EXPO-derived vesicles) are widely used as classical protein markers (Cai et al., 2023) (Figure 1A). Recent proteomic studies of apoplastic EVs from Arabidopsis and Brassica suggest that in addition to the well-established tetraspanins TET8 and TET9, additional marker families should be considered to fully capture the diversity of PDEV subtypes (Rodríguez De Lope et al., 2025).

By mining EV-enriched fractions from multiple plant species and comparing them to apoplastic soluble proteins, Rodríguez de Lope et al. proposed a broader panel of protein families with stronger evidence for EV specificity and phylogenetic conservation. These include aquaporins, vacuolar-type H^+^-ATPase subunits, selected fasciclin-like arabinogalactan proteins (FLAs), syntaxins (beyond PEN1), germin-like proteins and calreticulins, which are consistently enriched in genuine EV preparations and conserved across diverse plant taxa. Together with tetraspanins, these candidates provide a more versatile toolbox for future marker combinations, especially when aligned with Minimal Information for Studies of Extracellular Vesicles (MISEV)-style criteria (enrichment in vesicle fractions, depletion from non-vesicle fractions and conservation across datasets) (Welsh et al., 2024; Rodríguez de Lope et al., 2025). In parallel, functional cargo components, such as AGO-associated small RNAs and stress or defense-related proteins identified in apoplastic EVs, can serve as “contextual biomarkers” that report on specific PDEV subpopulations involved in immunity or environmental responses, complementing structural identity markers (Rutter and Innes, 2017; He et al., 2021; Koch et al., 2025).

### 4.2 Compositional signatures and biomarkers of PDNPs

In contrast to PDEVs, which are defined by conserved protein markers associated with their biogenesis pathways (e.g., PEN1, TET8, and FLAs) (Cai et al., 2023; Davila and He, 2025), PDNPs lack a universal protein marker. Because PDNPs are formed through the mechanical disruption of plant tissues, their composition is primarily a reflection of the bulk biomass from which they originate. Increasing evidence suggests that lipids, metabolites, and RNAs constitute the most reproducible and functionally informative classes of PDNP biomarkers, and that PDNP identity is better defined by dominant cargo composition and biological activity rather than by vesicle-associated proteins.

Lipids are among the most consistently reported PDNP components. PDNPs are enriched in phospholipids, glycolipids, sphingolipids, and fatty acids, with lipid composition varying substantially across plant species. For instance, ginger-derived nanoparticles (GDNPs) are characterized by a high enrichment of phosphatidic acid (PA), which accounts for nearly half of their total lipid content and is essential for their uptake by intestinal bacteria and mammalian cells (Zhang et al., 2016; Sundaram et al., 2019). Grapefruit-derived nanovesicles exhibit a different profile, dominated by phosphatidylethanolamine (PE) (45.52%) and phosphatidylcholine (PC) (28.53%) (Wang et al., 2014). Ginseng-derived PDNPs are enriched in diacylglycerol species associated with immune modulation, while celery-derived PDNPs contain abundant fatty acids such as linoleic acid that contribute to anti-inflammatory effects (Cho et al., 2021; Taşlı, 2022).

PDNPs frequently carry high levels of plant secondary metabolites, including flavonoids, phenolics, glucosinolates, and terpenoids, which are often enriched within or associated with nanoparticle membranes. Examples include naringin in grapefruit-derived PDNPs (Wang et al., 2014), sulforaphane in broccoli-derived PDNPs (Deng et al., 2017), and 6-gingerol and 6-shogaol in ginger-derived PDNPs (Zhang et al., 2016). These metabolites largely define the biological activities of PDNPs, such as anti-inflammatory, antioxidant, and antitumor effects, and provide strong species-specific signatures suitable for PDNP classification.

RNAs, particularly plant microRNAs, further contribute to PDNP functional identity. PDNP-associated microRNAs have been shown to regulate gene expression in recipient mammalian cells and gut microbiota, mediating interspecies communication. For example, ginger-derived PDNPs are enriched in specific microRNAs such as mdo-miR7267-3p, which can be taken up by gut bacteria and modulate microbial gene expression linked to intestinal homeostasis (Teng et al., 2018). Similarly, PDNPs derived from garlic contain abundant microRNAs, including miR396e, that have been implicated in regulating inflammatory pathways in mammalian cells (Liu et al., 2023). To provide a systematic classification of these diverse signatures, the key compositional markers and associated biological functions for major edible PDNPs are summarized in Table 1.

**TABLE 1:** PDNP biomarkers classified by cargo type and plant species.

Cargo type	Plant species	Markers/Cargoes	Biological function	References
Lipids	Ginger	Phosphatidic acid (PA)	Gut bacterial uptake; anti-inflammatory effects	Zhang et al. (2016), Sundaram et al. (2019)
	Grapefruit	Phosphatidylethanolamine (PE); Phosphatidylcholine (PC)	Immune modulation; mammalian cell targeting	Wang et al. (2014)
	Ginseng	Diacylglycerol; ceramides	Macrophage polarization; immune regulation	Cho et al. (2021)
	Celery	Fatty acids (e.g., linoleic acid)	Anti-inflammatory immune modulation	Taşlı (2022)
Metabolites	Grapefruit	Naringin; naringenin	Anticancer and immunomodulatory effects	Wang et al. (2014)
	Broccoli	Sulforaphane	AMPK activation; gut immune regulation	Deng et al. (2017)
	Ginger	6-gingerol; 6-shogaol	Colitis attenuation; intestinal repair	Zhang et al. (2016)
	Tea leaf	Polyphenol and flavonoid	Induce apoptosis of tumor cells	Chen et al. (2023)
	Cannabis	Cannabidiol	Induce cell death in hepatocellular carcinoma cell lines	Liu et al. (2022a)
miRNAs	Ginger	miR7267-3p	Gut microbiota modulation; anti-inflammatory signaling	Teng et al. (2018)
	Garlic	miR396e	Immune regulation; suppression of inflammatory pathways	Liu et al. (2023)
	Broccoli	miR167a	Induce apoptosis in pancreatic cancer cells	Wang et al. (2023)

## 5 Functional roles and application potential of PDEVs and PDNPs

### 5.1 Functional roles and application potential of PDEVs

In their native context, PDEVs are integral components of plant immunity rather than passive by-products of stress. Arabidopsis TET8-positive exosomes accumulate at infection sites and deliver host small RNAs into *B. cinerea*, where they silence virulence genes involved in vesicle trafficking and cell wall remodeling, thereby restricting disease progression (Cai et al., 2018). More recent work shows that PDEVs can also transport host mRNAs into plant fungal pathogen *B. cinerea*, providing an additional layer of cross-kingdom “RNA warfare” in which plant transcripts can be translated into proteins in fungal cells and suppress fungal virulence (Wang S. et al., 2024). Independently, EVs isolated from sunflower apoplastic fluids have been shown to be taken up by fungal spores, causing severe growth inhibition, altered morphology, and cell death (Regente et al., 2009). Root-secreted EVs from crops such as *Solanum lycopersicum* and *Sorghum bicolor* also display broad antifungal activity against fungal pathogens, including *Fusarium oxysporum, B. cinerea* and *Alternaria alternata,* and are enriched in defense-related proteins, enzymes and secondary metabolites (De Palma et al., 2020). Together, these studies support a model in which PDEVs act as directed carriers of antimicrobial cargoes, small RNAs, mRNAs, proteins and metabolites that move across the plant microbe interface to suppress infection.

These natural functions position PDEVs as promising platforms for next-generation crop protection strategies. Cross-kingdom RNA trafficking already underlies two major RNA-based disease-control approaches: host-induced gene silencing (HIGS) and spray-induced gene silencing (SIGS), but both could be substantially improved by leveraging PDEV biology. For HIGS, transgenic plants produce dsRNAs or sRNAs that silence pathogen genes during infection. Recent identification of plant RNA-binding proteins that mediate selective sRNA loading into EVs provides a mechanistic framework to enhance HIGS efficacy (He et al., 2021). By engineering plants to increase EV biogenesis or strengthen the recruitment of silencing RNAs into vesicles, future HIGS strategies could achieve more efficient and durable RNA transfer into pathogens. For SIGS, externally applied RNAs rely on uptake by pathogens from the leaf surface. Using PDEVs, or EV-inspired synthetic vesicles, as carriers could protect sprayed RNAs from degradation, improve stability on plant surfaces, and enhance uptake by fungal pathogens, which naturally internalize plant EVs during infection (Qiao et al., 2023). EV-based or EV-mimic formulations may therefore require lower doses, reduce off-target effects, and provide more persistent delivery than naked RNA sprays.

Beyond crop protection, the same principles highlight the broader relevance of PDEVs across One Health. Many fungal pathogens that infect humans and animals share conserved RNA-uptake pathways with plant pathogenic fungi, suggesting that PDEV-inspired vesicles could be engineered as biocompatible carriers for antifungal RNAs or small-molecule therapeutics (Wytinck et al., 2020; Bruch et al., 2022; Liu et al., 2025). Their natural ability to cross fungal cell walls and deliver functional cargo makes them an attractive template for developing safer and more targeted antifungal delivery systems (Casadevall et al., 2009; Lai et al., 2023). Although the development of direct medical use of PDEVs remains in early stages, their stability, low toxicity, and intrinsic capacity for cross-kingdom communication point to significant potential for future translational applications in both agriculture and human health.

### 5.2 Functional roles and application potential of PDNPs

Although their origins differ fundamentally from PDEVs, PDNPs have demonstrated a wide range of biological functions, and their safety, abundance, and oral stability have made them attractive candidates for translational applications.

A major functional theme emerging across multiple plant species is immunomodulation and anti-inflammatory activity. Ginger-derived PDNPs enriched in phosphatidic acid and gingerols reduce colitis severity, modulate macrophage cytokine responses, and promote intestinal epithelial repair (Zhuang et al., 2015; Zhang et al., 2016; Chen et al., 2019). Grapefruit-derived nanoparticles exhibit anti-inflammatory effects in gut epithelial cells and can be engineered to carry therapeutic RNAs or small molecules (Mu et al., 2014; Wang et al., 2014). Broccoli- and cauliflower-derived PDNPs modulate immune signaling pathways and have shown benefits in gut, liver, and metabolic disorders (Deng et al., 2017). These immunomodulatory properties appear to arise from tissue-specific lipids and secondary metabolites that are naturally embedded in the nanoparticle membrane.

PDNPs have also shown strong potential as oral delivery systems, largely due to their natural stability in the gastrointestinal environment. Citrus, grape, ginger, and coconut PDNPs are efficiently taken up by intestinal epithelial cells and macrophages, protect encapsulated cargo from enzymatic degradation, and display preferential trafficking to gut, liver, or immune tissues depending on lipid composition (Raimondo et al., 2019; Elsharkasy et al., 2020). This has enabled the delivery of siRNAs, miRNAs, chemotherapeutics, anti-inflammatory compounds, and microbial-targeting agents in mouse models (Del Pozo-Acebo et al., 2022; Itakura et al., 2023). Several studies demonstrate that PDNPs enhance oral bioavailability and reduce systemic toxicity compared with conventional formulations (Wang et al., 2013; Zhang et al., 2016).

Another important area of investigation is microbiome modulation. Edible PDNPs from ginger, garlic, lemon, grapefruit, grape, and cruciferous vegetables can selectively alter microbial growth, reshape gut microbial composition, and influence microbial metabolites such as short-chain fatty acids or tryptophan derivatives (Teng et al., 2018; Del Pozo-Acebo et al., 2021; Chen et al., 2022; Liu Y. et al., 2022; Niu et al., 2023; Wang X. et al., 2024). This microbiota-targeting activity supports therapeutic applications in gastrointestinal disorders, metabolic disease, neuroinflammation, and mucosal immunity.

Collectively, PDNPs represent a versatile class of plant-derived nanomaterials with broad application potential in human health, microbiome engineering, and oral drug delivery. Their biological activities arise not from selective biogenesis but from the rich lipid and metabolite composition of plant tissues, which gives them unique advantages for therapeutic delivery, nutritional supplementation, and modulation of host–microbe interactions.

## 6 Discussion and conclusion

Although PDEVs and PDNPs are often discussed together, this review highlights that they arise from fundamentally different biological origins. PDEVs are actively secreted nanovesicles produced through regulated pathways and enriched with selectively loaded cargo, whereas PDNPs are generated through tissue disruption and reflect the intrinsic lipid-metabolite composition of plant biomass. Clarifying this distinction is essential for establishing standardized isolation methods, defining appropriate biomarkers, and improving reproducibility across studies.

Each system offers unique strengths for translational applications. PDEVs have a stronger mechanistic foundation, supported by detailed work showing selective RNA loading, targeted delivery into fungal pathogens, and defined roles in plant immunity. These insights into plant-pathogen trafficking offer broader lessons for cross-kingdom RNA communication that may extend to human and animal pathogenic fungi, suggesting opportunities to design biomimetic antifungal carriers inspired by PDEV biology. PDNPs, in contrast, have advanced more rapidly toward applied use, particularly in mammalian systems. Their stability, scalability, safety, and ability to deliver metabolites or therapeutic cargo have made them promising candidates for oral drug delivery, microbiome modulation, and immunotherapy. Together, PDEVs contribute mechanistic rigor, while PDNPs offer practical advantages for translation.

Looking forward, both PDEVs and PDNPs hold potential for agricultural and One Health applications. PDEVs naturally mediate RNA and protein movement between plants and microbes, providing a biological template for developing next-generation RNA delivery technologies such as enhanced HIGS or SIGS systems. PDNPs, owing to their abundance, biocompatibility, and low toxicity, are increasingly recognized as appealing candidate platforms for sustainable agricultural delivery systems, particularly for RNA-based crop protection strategies and the transport of bioactive molecules. At present, their use for delivering conventional agrochemicals such as pesticides or fertilizers remains at an early stage, as direct plant-level validation is still limited. Thus, current studies primarily position PDNP-based delivery as an emerging research direction, highlighting the need for further experimental investigation. Key challenges remain, including the need for validated biomarkers, greater methodological consistency, improved mechanistic understanding, and clearer distinctions between particle types, but addressing these gaps will be essential for transforming plant-derived nanovesicles into reliable, application-ready platforms across biotechnology, agriculture, and medicine.
